# A Three-Dimensional Bioabsorbable Tissue Marker for Volume Replacement and Radiation Planning: A Multicenter Study of Surgical and Patient-Reported Outcomes for 818 Patients with Breast Cancer

**DOI:** 10.1245/s10434-020-09271-2

**Published:** 2020-11-21

**Authors:** Cary S. Kaufman, Michael J. Cross, Julie L. Barone, Nayana S. Dekhne, Kiran Devisetty, Joshua T. Dilworth, David A. Edmonson, Firas G. Eladoumikdachi, Jennifer S. Gass, William H. Hall, Robert L. Hong, Robert R. Kuske, Brandon J. Patton, Carol Perelson, Rogsbert F. Phillips, Arnold B. Smith, Linda A. Smith, Lorraine Tafra, Gail S. Lebovic

**Affiliations:** 1 Department of Surgery, University of Washington, Seattle, WA USA; 2 Bellingham Regional Breast Center, 2075 Barkley Blvd. Suite 250, Bellingham, WA USA; 3 Breast Treatment Associates, Fayetteville, AR USA; 4 Saint Joseph Hospital, Denver, CO USA; 5 Beaumont Hospitals, Southfield, MI USA; 6 Karmanos Cancer Institute, Detroit, MI USA; 7 William Beaumont Hospital, Royal Oak, MI USA; 8 Womens Oncology, Women and Infants Hospital, Providence, RI USA; 9 Women and Infants Hospital, Breast Health Center, Providence, RI USA; 10 Radiation Oncology, PeaceHealth St. Joseph Medical Center, Bellingham, WA USA; 11 Virginia Hospital Center, Arlington Health System, Arlington, VA USA; 12 Arizona Breast Cancer Specialists, Scottsdale, AZ USA; 13 New York, NY USA; 14 Metro Surgical Associates, Emory Healthcare, Atlanta, GA USA; 15 Highlands Oncology Group, Fayetteville, AR USA; 16 Comprehensive Breast Care, Albuquerque, NM USA; 17 Anne Arundel Medical Center, The Breast Center, Annapolis, MD USA; 18 School of Oncoplastic Surgery, Reno, NV USA

## Abstract

**Background:**

Accurate identification of the tumor bed after breast-conserving surgery (BCS) ensures appropriate radiation to the tumor bed while minimizing normal tissue exposure. The BioZorb^®^ three-dimensional (3D) bioabsorbable tissue marker provides a reliable target for radiation therapy (RT) planning and follow-up evaluation while serving as a scaffold to maintain breast contour.

**Methods:**

After informed consent, 818 patients (826 breasts) implanted with the BioZorb^®^ at 14 U.S. sites were enrolled in a national registry. All the patients were prospectively followed with the BioZorb^®^ implant after BCS. The data collected at 3, 6, 12, and 24 months included all demographics, treatment parameters, and provider/patient-assessed cosmesis.

**Results:**

The median follow-up period was 18.2 months (range, 0.2–53.4 months). The 30-day breast infection rate was 0.5 % of the patients (*n* = 4), and re-excision was performed for 8.1 % of the patients (*n* = 66), whereas 2.6 % of the patients (*n* = 21) underwent mastectomy. Two patients (0.2 %) had local recurrence. The patient-reported cosmetic outcomes at 6, 12, and 24 months were rated as good-to-excellent by 92.4 %, 90.6 %, and 87.3 % of the patients, respectively and similarly by the surgeons. The radiation oncologists reported planning of target volume (PTV) reduction for 46.2 % of the patients receiving radiation boost, with PTV reduction most commonly estimated at 30 %.

**Conclusions:**

This report describes the first large multicenter study of 818 patients implanted with the BioZorb^®^ tissue marker during BCS. Radiation oncologists found that the device yielded reduced PTVs and that both the patients and the surgeons reported good-to-excellent long-term cosmetic outcomes, with low adverse effects. The BioZorb^®^ 3D tissue marker is a safe adjunct to BCS and may add benefits for both surgeons and radiation oncologists.

**Electronic supplementary material:**

The online version of this article (10.1245/s10434-020-09271-2) contains supplementary material, which is available to authorized users.

Breast-conserving surgery (BCS) with radiotherapy has demonstrated equivalent or better survival outcomes compared with mastectomy.^1^^–^10 Because oncologic outcomes have improved and patients are living longer, the cosmetic result of surgery has gained increasing clinical importance as an additional parameter of surgical quality. The significant relationship between patient perception of breast cosmesis with quality of life and psychosocial functioning also has been established.^11^^–^14 Long-term cosmetic outcomes with BCS may be suboptimal due to delayed surgical scarring and radiation effects, with a significant proportion of patients unhappy about their breast cosmesis after BCS and adjuvant radiation therapy (RT).^15^

The risk of poor cosmetic outcome is increased for patients with large or medially located tumors.^16^^–^19 Two factors that have an impact on cosmesis are commonly identified. One factor is the loss of the natural breast contour after tumor excision, with an inverse relationship observed between the amount of excised breast volume and patient satisfaction with breast cosmesis.^16^,^20^ The other factor is RT-induced tissue damage, with a recognized correlation between the targeted volume of breast radiation and worsening post-RT breast cosmesis.^21^^–^26 By providing a three-dimensional (3D) indicator within the surgical excision site, surgeons may enhance communication with radiation oncologists (ROs) for more precise targeting of the tumor bed, which may allow reduction in tumor bed boost volumes.

Accurate identification of the tumor bed after BCS can be technically challenging due to seroma formation at both the surgical site and the areas of tissue mobilization and rearrangement. Remote incisions may add further confusion for ROs while improving immediate cosmetic outcome.^27^^–^31 Surgical clips traditionally used for this purpose have several known limitations including clip movement relative to breast position, confusion with clips used for hemostasis, and risk of underdosing while clip location is being interpreted.^27^,^32^,^33^

Often, ROs must interpret clip placement in tandem with traditional methods to avoid inaccurate estimation of treatment volume or even a geographic miss.^28^,^34^ To avoid undertreatment due to lack of clarity between cancer surgical areas and tissue rearrangements, more generous planning target volume (PTV) margins often are used. This, in turn, can lead to increased radiation side effects of fat necrosis, breast firmness, and poor cosmetic outcome.^23^,^35^

One proposed tool for marking the surgical site is the BioZorb^®^ 3D bioabsorbable marker (Focal Therapeutics, Sunnyvale, CA, USA [Focal Therapeutics was acquired by Hologic, Inc., Marlborough, Massachusetts in October 2018]). Positioned at the tumor excision site with several sutures, the open structure with six titanium clips spatially oriented provides a 3D target visible from any angle. The marker serves both as a scaffold for tissue rearrangement and oncoplastic closure, and also as a mild volume replacement for the excised tissue. Though the framework eventually resorbs after tissue healing, the six affixed radiopaque clips remain in position for future imaging. Re-excision orientation is facilitated because each margin is identified by its attachment to the 3D marker. This marker has been associated with positive cosmetic and overall patient outcomes as well as reduced RT volumes in the few reported studies to date.^36^–^42^ These studies have been small, focused on RT planning outcomes or limited to short-term follow-up evaluation. This is the first report of 24-month outcomes after BCS with BioZorb^®^ (BZ) implantation in a large U.S. registry of data collected across an array of clinical settings.

## Methods

### Study and Device Design

The BioZorb^®^ Registry collected data at 14 U.S. sites in 13 states, with 42 surgeons and 76 ROs participating in the study. The study considered women 18 years of age or older who were breast cancer candidates for BCS as eligible for enrollment. The exclusion criteria ruled out a history of breast cancer or radiation in the same breast, multifocal breast cancer, and breast implants. A full list of the inclusion and exclusion criteria is available in Table S1.

All the patients provided informed consent before participation in the registry. The patients enrolled prospectively all provided informed consent before BCS with BZ implantation. The patients enrolled retrospectively after BCS with BZ implantation provided informed consent before chart review, with charts prospectively followed from the time of informed consent. This group was enrolled before their first post-surgery visit. At all study sites, institutional review board approval was obtained, and the study was conducted in accordance with the Declaration of Helsinki.

**Fig. 1:**
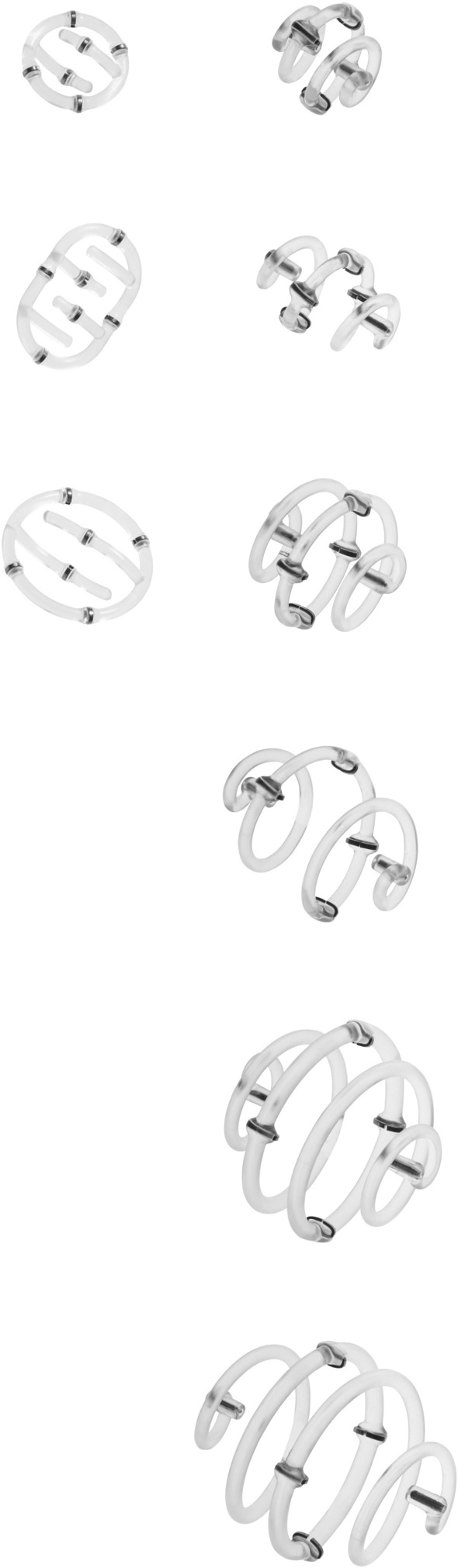
The BioZorb^®^ three-dimensional (3D) bioabsorbable tissue marker. The 3D BioZorb^®^ configuration is available in six sizes (2 × 2 cm, 2 × 3 cm, 3 × 3 cm, 3 × 4 cm, 4 × 4 cm, and 4 × 5 cm). A low-profile design was released in 2015 (left), with sizes ranging from 2 to 3 cm by 1 cm deep

The BZ device has a bioabsorbable spiral structure composed of polylactic acid and six small embedded titanium clips. The BZ is available in a range of sizes and configurations, including a low-profile design to allow tailoring of the device to the surgical cavity (Fig. 1). Additional details on device design and its appearance on several imaging methods have been published previously.^43^

### Study Procedure and Follow-up Schedule

All the enrolled patients underwent BCS as treatment for breast cancer, with the BZ positioned at the tumor bed margins. Candidates for BZ placement were decided by individual surgeons based on clinical presentation. All medical management was at the discretion of the individual surgeon.

General recommendations for BZ placement were available to surgical users. A sizer set was provided with the device, with the selection of BZ size and shape guided by the size and shape of the tumor rather than by matching the larger lumpectomy cavity.

At least three sutures made of monofilament absorbable suture were used to secure the BZ to the walls of the lumpectomy cavity. Local breast reconstruction with oncoplastic techniques was per the operating surgeon’s practice. Surgeons classified the cavity closure performed as no cavity closure, minor mobilization of local tissue flaps, or moderate-to-extensive tissue rearrangement.

Surgical follow-up evaluation comprised visits at 3, 6, 12, 18, 24, and 36 months. For the purpose of data analysis, surgical follow-up visit intervals post BCS were defined as follows: 0–135 days (3 months), 136–270 days (6 months), 271–450 days (12 months), 451–630 days (18 months), 631–900 days (24 months), and 901–1260 days (36 months).

### Data Collection and Statistical Analysis

The registry data collected included patient demographics and clinicopathologic characteristics, operative details, RT planning characteristics, adverse effects (AEs), and reoperations through follow-up evaluation. Additionally, patients, surgeons, and ROs rated breast cosmesis at the time of surgery and at all follow-up visits. Surgeons and ROs also rated device impact on utility, visibility, motivation for usage, and cosmesis. All AEs were captured whether they were symptomatic or not. The AEs were categorized and entered into the database by investigators regardless of the detection method or whether they required treatment. Date of onset, date of resolution, and location within the breast were captured for all AEs. The AEs labeled as “other” were manually reviewed to ensure that all AEs belonging to one of the specified categories (e.g., breast infection) were identified and categorized appropriately.

Reintervention counts comprised events occurring through all follow-up visits. Tabular reporting of AEs together with patient and provider responses at each interval was through 24-month visit windows because only 149 patients (18.2 %) had 36-month follow-up data available as of October 2019.

The percentages listed were calculated with denominators comprising the number of patients (or breasts, dependent on data point) with available data for a given data point. An October 2019 data export provided the basis for the current report. The SAS programming of study data was performed by BioStat International (Tampa, FL, USA).

## Results

### Study Population and Treatment Characteristics

From July 2012 through August 2019, 818 patients underwent BCS with BZ implantation. Eight of these patients (1 %) were bilaterally treated, for a total of 826 BZ implantations.

The baseline demographic and clinicopathologic characteristics are listed in Table 1. The average tumor size was 15.4 mm (range, 0–125 mm). The distribution of the breast cancers showed 65.1 % to be invasive ductal carcinoma, 8.4 % to be invasive lobular carcinoma, 20.2 % to be ductal carcinoma in situ, and 6.2 % to be other.

**Table 1:** Demographic and clinicopathologic characteristics of 826 tumors treated in 818 patients^a^

Characteristic	*n* (%)
Median age: years (range) (n = 818)	63.0 (24.6–87.7)
<40	14 (1.7)
40–49	88 (10.8)
50–59	230 (28.1)
≥60	486 (59.4)
Race (*n* = 810)	
Caucasian	691 (85.3)
Black or African American	99 (12.2)
Asian	12 (1.5)
AI/AN/NH/PI	8 (1.0)
No. of patients bilaterally treated	8 (1.0)
Breast side (*n* = 826)	
Left	427 (51.7)
Right	399 (48.3)
Breast quadrant (*n* = 819)	
Upper outer	403 (49.2)
Upper medial	193 (23.6)
Lower outer	129 (15.8)
Lower medial	94 (11.5)
Cup size (*n* = 469)	
A	21 (4.5)
B	126 (26.9)
C	155 (33.0)
D	88 (18.8)
>D	79 (16.8)
Histology (*n* = 822)	
DCIS	166 (20.2)
IDC	535 (65.2)
ILC	69 (8.4)
Other	52 (6.3)
Median tumor size: mm (range) (*n* = 809)	15.4 (0–125)
≤20	625 (77.3)
>20	184 (22.7)
AJCC tumor (*n* = 811)	
Benign	16 (2.0)
Tis	150 (18.5)
T1a	76 (9.4)
T1b	153 (18.9)
T1c	265 (32.6)
T2	139 (17.3)
T3	12 (1.5)
Node-positive (*n* = 821)	122 (14.9)
ER-positive (*n* = 806)	692 (85.9)
PR-positive (*n* = 791) HER2-positive (*n* = 653)	603 (76.2) 71 (10.9)

*AI* American Indian; *AN* Alaska native; *NH* native Hawaiian; *PI* Pacific Islander; *DCIS* ductal carcinoma in situ; *IDC* invasive ductal carcinoma; *ILC* invasive lobular carcinoma; *AJCC* American Joint Committee on Cancer; *ER* estrogen receptor; *PR* progesterone receptor; *HER2* human epidermal growth factor receptor 2

^a^Continuous data are reported as median (range) and categorical data as number (%)

The operative details are listed in Table 2. The device sizes most commonly implanted were 2 × 2 cm (37.5 %) and 2 × 3 cm (35.6 %). The low-profile BZ configurations that enabled increased use in A cup breasts and other thin areas of the breast did not become available until August 2015. Moderate or extensive tissue mobilization for cavity closure was performed for 55.8 % of the treated breasts (*n* = 445), and minor mobilization of surrounding tissue flaps for cavity closure was performed for 40.7 % of the treated breasts (*n* = 325).

**Table 2:** Operative details^a^

Characteristic	*n* (%)
Device-sizer set used (*n* = 791)	599 (75.7)
Device size (*n* = 784)	
2 cm × 2 cm	294 (37.5)
2 cm × 3 cm	279 (35.6)
3 cm × 3 cm	113 (14.4)
3 cm × 4 cm	62 (7.9)
4 cm × 4 cm	19 (2.4)
4 cm × 5 cm	17 (2.2)
Low profile (*n* = 703)^b^	44 (6.3)
Device sutured into position (*n* = 819)	818 (99.9)
No. of sutures to secure device into position (*n* =790)	
1–3	528 (66.8)
>3	262 (33.2)
Reported purpose for BZ usage (*n* = 631)	
Communication tool with radiation oncology	577 (91.4)
Help to improve cosmetic appearance	394 (62.4)
Marking tumor excision site	337 (53.4)
Scaffolding for oncoplastic closure techniques	311 (49.3)
Assistance with locating area for re-excision	238 (37.7)
No. with intraoperative sentinel node examination (n = 823)	687 (83.5)
No. of sentinel nodes examined	2 (0–15)
No. with positive nodes (*n* = 560)	97 (17.3)
No. with axillary dissection (*n* = 823)	58 (7.0)
No. with chemotherapy (*n* = 826)	28 (3.4)
No. with hormone therapy (*n* = 826)	54 (6.5)

*BZ* BioZorb^®^

^a^Categorical data are reported as number (%). Data are reported per treated breast

^b^Use of the low profile (LP) device was not specifically captured in the registry; however, the surgeons indicated use of the LP device with device lot numbers for 44 patients

**Fig. 2:**
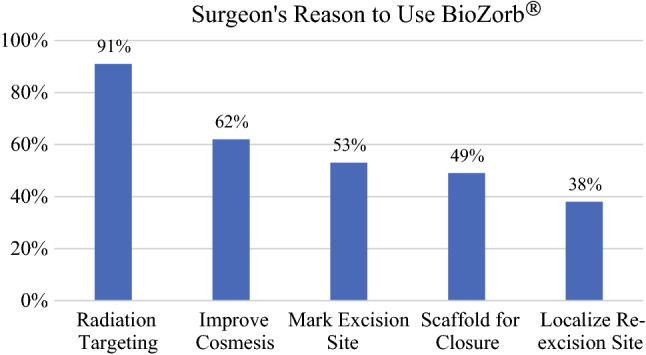
Surgeons’ reported purpose for three-dimensional (3D) tissue marker use. Reasons why the surgeons stated that they used the 3D bioabsorbable marker (*n* = 631). Multiple reasons may be reported

The reason for BZ implantation by surgeons was provided in 76.4 % of the cases (*n* = 631; Fig. 2). The main reasons identified by surgeons to use the BZ were communication with the RO in 577 cases (91.4 %), followed by the desire to improve the cosmetic outcome in 394 cases (62.4 %).

### Radiation Therapy Characteristics

Of the 818 patients in the registry, 81 (9.9 %) were not associated with any RO inside or outside the registry. In addition, radiation was not administered to 11 patients (1.3 %) due to either physician recommendation (*n* = 7) or refusal (*n* = 4). For the remaining 726 patients listed as having an RO (32 of whom had an outside-of-registry RO), registry data were submitted by the RO for 484 patients (69.7 %) and 488 breasts. Of the 488 cases with available RO survey responses on usefulness of the BZ for RT case planning, 73.6 % (*n* = 359) were managed via whole-breast radiation with a boost, and 12.7 % (*n* = 62) were managed via whole-breast radiation without a boost. Of the cases with data on the type of RT provided, 56.5 % (*n* = 255) were managed with hypofractionated whole-breast irradiation (HF-WBI), and 36.7 % (*n* = 166) received conventional whole-breast irradiation (WBI). Accelerated partial-breast irradiation (APBI) was administered to 64 patients (13.1 %), with 3 patients having missing RT type data.

Radiation oncologists marked the visibility of the BZ as “easily seen” on computed tomography (CT) in 94.8 % of the cases overall and in 100 % of the APBI cases. The RO survey responses on primary treatment planning are presented in Table 3. Because the RO response rate varied from question to question, these variations are reflected in the denominators.

**Table 3:** Utility of BioZorb^®^ (BZ) for primary radiation therapy treatment planning^a^

Primary radiation therapy (RT) treatment planning	All breasts (*n* = 488)^b^ *n* (%)	All WBI/HypoFx with boost (*n* = 359) *n* (%)	All WBI/HypoFx without boost (*n* = 62) *n* (%)	APBI (*n* = 64) *n* (%)
Visibility of BZ marker clips on CT	(*n* = 462)	(*n* = 348)	(*n* = 49)	(n = 63)
Easily seen	438 (94.8)	326 (93.7)	47 (95.9)	63 (100.0)
Noticeable	23 (5.0)	21 (6.0)	2 (4.1)	0 (0.0)
Poorly seen	1 (0.2)	1 (0.2)	0 (0.0)	0 (0.0)
Not visible	0 (0.0)	0 (0.0)	0 (0.0)	0 (0.0)
Usefulness of BZ for primary treatment planning	(*n* = 451)	(*n* = 347)	(*n* = 49)	(*n* = 53)
Very useful	88 (19.5)	31 (8.9)	10 (20.4)	46 (86.8)
Fairly useful	91 (20.2)	70 (20.2)	15 (30.6)	6 (11.3)
Somewhat useful	77 (17.1)	64 (18.4)	12 (24.5)	1 (1.9)
Not useful	195 (43.2)	182 (52.4)	12 (24.5)	0 (0.0)
Usefulness of BZ for reducing size of PTV	(*n* = 447)	(*n* = 348)	(*n* = 49)	(*n* = 48)
Yes	133 (29.8)	106 (20.5)	6 (12.2)	20 (41.7)
No	155 (34.7)	116 (33.3)	29 (59.2)	10 (20.8)
N/A	159 (35.6)	126 (36.2)	14 (28.6)	18 (37.5)
If yes, approximate PTV volume reduction (%)	(*n* = 130)	(*n* = 105)	(*n* = 4)	(*n* = 20)
10	18 (13.8)	17 (16.2)	0 (0.0)	1 (5.0)
20	24 (18.5)	14 (13.3)	2 (50.0)	7 (35.0)
30	84 (64.6)	73 (69.5)	1 (25.0)	10 (50.0)
40	4 (3.1)	1 (0.9)	1 (25.0)	2 (10.0)
≥50	0 (0.0)	0 (0.0)	0 (0.0)	0 (0.0)
BZ helpful in planning of HypoFx	(*n* = 403)	(*n* = 306)	(*n* = 36)	(*n* = 59)
Very helpful	31 (7.7)	6 (2.0)	5 (13.9)	19 (32.2)
Fairly helpful	18 (4.5)	11 (3.6)	6 (16.7)	1 (1.7)
Somewhat helpful	35 (8.7)	25 (8.2)	10 (27.8)	0 (0.0)
Not helpful	52 (12.9)	47 (15.4)	4 (11.1)	1 (1.7)
N/A	267 (66.3)	217 (70.9)	11 (30.6)	38 (64.4)
BZ used for patient setup	(*n* = 462)	(*n* = 348)	(*n* = 49)	(*n* = 63)
Yes	95 (20.6)	56 (16.1)	11 (22.4)	27 (42.9)
No	367 (79.4)	292 (83.9)	38 (77.6)	36 (57.1)

*WBI* whole-breast irradiation; *HypoFx* hypofractionated radiotherapy; *APBI* accelerated partial breast irradiation; *CT* computed tomography; *PTV* planning target volume; *N/A* not applicable for radiation therapy treatment planning

^a^Categorical data are reported as number (%). Data are reported per treated breast

^b^In 3 cases with radiation oncologists survey responses, type of RT provided was not specified and is not included in the RT subgroup breakdown

Overall, ROs reported that BZ was useful for RT planning for 56 % of the patients, which depended on whether focused RT (boost or partial-breast irradiation) was performed. The ROs planning to deliver APBI rated the device highest, with the BZ considered very useful in 86.8 % of cases. The ROs were asked whether the BZ was useful in reducing the size of the PTV for primary treatment planning. When the question was considered applicable by the RO, the BZ was deemed useful for reducing the PTV in 46.2 % of cases receiving whole-breast radiation (hypofractionated or conventional) with boost and 66.7 % of cases receiving APBI. For the cases in which the BZ was considered useful for PTV reduction, the ROs were asked to approximate the PTV volume reduction. The ROs most commonly estimated a PTV reduction of 30 % (64.6 % of cases; Table 3). Regarding boost treatment specifically, when asked “Did BioZorb^®^ improve your accuracy in boost targeting?” ROs responded “yes” in 306 cases (87.4 %) and “no” in 44 cases (12.6 %) with boost survey data (Table 4). The BZ was useful for boost treatment planning for 96.6 % of the patients, with 69.8 % considering it “very useful.”

**Table 4:** Utility of BioZorb^®^ (BZ) for boost treatment planning

Radiation therapy (RT) boost treatment planning	All breasts with boost (*n* = 363)^a^ *n* (%)
Visibility of BZ marker clips on CT	(*n* = 352)
Easily seen	329 (93.5)
Noticeable	22 (6.3)
Poorly seen	1 (0.3)
Not visible	0 (0.0)
Usefulness of BZ for boost planning	(*n* = 351)
Very useful	245 (69.8)
Fairly useful	57 (16.2)
Somewhat useful	37 (10.5)
Not useful	12 (3.4)
Did BZ improve your accuracy for boost targeting?	(*n* = 350)
Yes	306 (87.4)
No	44 (12.6)
Was BZ helpful in planning field in field?	(*n* = 337)
Very helpful	18 (5.3)
Fairly helpful	10 (3.0)
Somewhat helpful	0 (0.0)
Not helpful	3 (0.9)
N/A	306 (90.8)
Was BZ used for patient setup?	(*n* = 350)
Yes	182 (52.0)
No	168 (48.0)
BZ used for patient setup	(*n* = 178)
EPID	29 (16.3)
CBCT	64 (36.0)
OBI	9 (5.1)
Other	76 (42.7)

*CT* computed tomography; *NA* not applicable for boost treatment planning; *EPID* electronic portal imaging device; *CBCT* cone-beam computed tomography; *OBI* on-board imaging

^a^In 3 cases with radiation oncologists survey responses, type of RT provided was not specified and is not included in the RT subgroup breakdown

### Follow-up and Adverse Effects

The median follow-up period was 18.2 months (range, 0.2–53.4 months). As of October 2019, 87.3 % of the patients (*n* = 714) still were enrolled in the registry or had completed the registry follow-up visits, and 12.7 % (*n* = 104) had left the registry. Of these patients, 6.6 % (*n* = 54) were lost to follow-up evaluation, 2.4 % (*n* = 20) had undergone a mastectomy, 0.7 % (*n* = 6) had died, 0.2 % (*n* = 2) had elected to withdraw, and 2.7 % (*n* = 22) had discontinued for other reasons (mostly unavailability for follow-up evaluation).

Adverse effects occurred in 3.8 % of the patients (*n* = 31) within 30 days after BCS. The AEs included seroma formation (1.9 %, *n* = 16), breast infection (0.5 %, *n* = 4), pain (0.5 %, *n* = 4), hematoma (0.2 %, *n* = 2), unspecified effects (0.2 %, *n* = 2), fat necrosis (0.1 %, *n* = 1), flap ischemia (0.1 %, *n* = 1), poor wound healing (0.1 %, *n* = 1), infected sentinel node incision (0.1 %, *n* = 1), and problematic palpability (0.1 %, *n* = 1). Some of the patients may have had more than one AE. Through the entire follow-up period, 17 (2.1 %) of the patients had breast infection and 27 (3.3 %) had seroma formation. Nearly 60 % of the infections occurred at a single institution (*n* = 10), with two infections occurring at another site, and a single infection occurring at five institutions. The institution with the most infections also was the largest accrual site, with more 250 patients in the study. Only 5 of the 17 patients noted to have an infection required removal of the devices during the study period, and the remaining 71 % of the devices remained in the breast after infections resolved. Full reporting of AEs by category and follow-up interval is presented in Table 5.

**Table 5:** Adverse effects (AEs) observed during the 24-month follow-up period^a^

	Overall *n* (%)	3-Month FU (0–135 days) (*n* = 826) *n* (%)	6-Month FU (136–270 days) (*n* = 656) *n* (%)	12-Month FU (271–450 days) (*n* = 569) *n* (%)	18-Month FU (451–630 days) (*n* = 433) *n* (%)	24-Month FU (631–900 days) (*n* = 337) *n* (%)
Breasts with any AE						
Overall	91 (11.0)	61 (7.4)	13 (2.0)	15 (2.6)	5 (1.2)	4 (1.2)
Symptomatic	74 (9.0)	54 (6.5)	9 (1.4)	9 (1.6)	4 (0.9)	3 (0.9)
Requiring any treatment^b^	81 (9.8)	58 (7.0)	10 (1.5)	13 (2.3)	4 (0.9)	2 (0.6)
Requiring invasive/narcotic/AB treatment^b^	49 (5.9)	36 (4.4)	8 (1.2)	7 (1.2)	2 (0.5)	0 (0.0)
AE (any grade)						
Seroma formation	27 (3.3)	24 (2.9)	2 (0.3)	1 (0.2)	1 (0.2)	0 (0.0)
Breast infection	17 (2.1)^c^	13 (1.6)^c^	1 (0.2)	2 (0.4)	1 (0.2)	0 (0.0)
Fat necrosis	2 (0.2)	1 (0.1)	0 (0.0)	0 (0.0)	0 (0.0)	1 (0.3)
Pain (any)	16 (1.9)	9 (1.1)	3 (0.5)	2 (0.4)	1 (0.2)	1 (0.3)
Telangiectasia	1 (0.1)^d^	0 (0.0)	0 (0.0)	0 (0.0)	0 (0.0)	0 (0.0)
Hypo-pigmentation	0 (0.0)	0 (0.0)	0 (0.0)	0 (0.0)	0 (0.0)	0 (0.0)
Hyper-pigmentation	7 (0.8)	3 (0.4)	2 (0.3)	2 (0.4)	0 (0.0)	0 (0.0)
Fibrosis	5 (0.6)	0 (0.0)	1 (0.2)	3 (0.5)	1 (0.2)	0 (0.0)
Other^e^	33 (4.0)	20 (2.4)	6 (0.9)	5 (0.9)	1 (0.2)	2 (0.6)
AE (grade 3 or higher)						
Telangiectasia	0 (0.0)	0 (0.0)	0 (0.0)	0 (0.0)	0 (0.0)	0 (0.0)
Pigmentation	1 (0.1)	0 (0.0)	1 (0.2)	0 (0.0)	0 (0.0)	0 (0.0)
Fibrosis	0 (0.0)	0 (0.0)	0 (0.0)	0 (0.0)	0 (0.0)	0 (0.0)
Pain intensity (*n* = 14)^f^						
1–3 (mild)	8 (1.0)	5 (0.6)	0 (0.0)	2 (0.4)	1 (0.2)	0 (0.0)
4–6 (moderate)	5 (0.6)	2 (0.2)	2 (0.3)	0 (0.0)	0 (0.0)	1 (0.3)
7–10 (severe)	1 (0.1)	1 (0.1)	0 (0.0)	0 (0.0)	0 (0.0)	0 (0.0)
AE location						
Arm	2 (0.2)	2 (0.2)	0 (0.0)	0 (0.0)	0 (0.0)	0 (0.0)
Axilla	10 (1.2)	6 (0.7)	2 (0.3)	1 (0.2)	0 (0.0)	1 (0.3)
Breast	83 (10.0)	55 (6.7)	12 (1.8)	15 (2.6)	5 (1.2)	4 (1.2)
Chest wall	3 (0.40)	3 (0.4)	0 (0.0)	0 (0.0)	0 (0.0)	0 (0.0)
Other	3 (0.40)	1 (0.1)	0 (0.0)	1 (0.2)	0 (0.0)	1 (0.3)

*FU* follow-up; *AB* antibiotic

^a^Categorical data are reported as number (%). Data are reported per treated breast.

^b^All treatments included nonsteroidal antiinflammatory drugs (NSAIDs), non-narcotic analgesics, observation, and other noninvasive treatments such as heat application, as well as invasive/narcotic/antibiotic treatment (presented below as a separate count)

^c^The 30- and 90-day breast infection rates were respectively 0.5 % (*n* = 4) and 1.3 % (*n* = 11)

^d^Telangiectasia observed at 36 months

^e^Other AEs observed included redness/rash/erythema/postradiation mastitis or injury (*n* = 9), poor wound healing/wound dehiscence (*n* = 6), device palpability (*n* = 3), hematoma (*n* = 2), and 1 each of flap ischemia, sentinel node incision infection, lymphedema, strange sensation, palpable nodule, and visible hard lump in lumpectomy bed thought to be BZ clip migration

^f^Pain intensity (pain from breast-conserving surgery [BCS] or BioZorb^®^ [BZ] not differentiated) was captured via a 10-point scale. Groupings of 1–3 (mild), 4–6 (moderate), and 7–10 (severe) are per the SRA Lab’s numeric pain rating scale, which was adopted from McCaffery M., Beebe A., et al. (1989). *Pain: Clinical Manual for Nursing Practice.* Mosby St. Louis, MO). Highest pain level recorded was a 7 (*n* = 1), and lowest pain recorded was a 2 (*n* = 3). One patient had missing pain-intensity evaluation (pain event occurring within a 3-month window)

### Reoperation and Device Removal After BCS

Re-excision for positive margins was performed for 8.1 % of the patients (*n* = 66) during the entire follow-up period, with 1 % (*n* = 8) requiring two re-excisions. The re-excision rates varied widely across the 14 study centers, with a median re-excision rate of 4.9 % (range, 0.0 %–19.8 %). During the follow-up period, 2.6 % of the patients (*n* = 21) underwent mastectomy. The indications for mastectomy were positive margins (*n* = 7), multifocal disease (*n* = 3), disease recurrence (*n* = 2), patient choice (*n* = 2), bilateral prophylactic mastectomy (*n* = 2), and unknown reasons (*n* = 5). The rates of conversion to mastectomy were low at all the centers (median, 1.5 %; range, 0.0 %–6.3 %).

Except for mastectomies, the BZ device was removed from 2.7 % of the patients (*n* = 22) during the entire follow-up period. The BZ was removed for the following circumstances: re-excision for margins (*n* = 10), infection (or complications resultant from infection; *n* = 5), patient request due to anxiety or pain (*n* = 2), local recurrence (*n* = 1), removal of microcalcifications (*n* = 1), and unknown reasons (*n* = 3).

### Local and Distant Recurrence

From 2012 through 2019, 0.5 % of the patients (*n* = 4) experienced cancer recurrence (2 local, 2 distant). For one patient, the original cancer was multifocal, and local recurrence involved an undiagnosed separate satellite in the same breast. Recurrence was detected 2 years after BCS and treated with a second lumpectomy, with the original device left intact. Another patient with local recurrence had a 2.5-cm ductal carcinoma in situ (DCIS) and chose not to undergo radiation. Recurrence was observed 21 months after BCS, followed with a subsequent mastectomy. One patient with distant recurrence had a 2.1-cm invasive ductal carcinoma (IDC) and experienced brain metastasis. This tumor was detected 13 months after the initial BCS, and the patient died 19 months after the BCS. One patient who had IDC with multiple positive lymph nodes experienced metastasis to the bones. This was detected at 6 months, and the patient died 10 months after the BCS.

### Breast Cosmesis

The patient and surgeon cosmesis assessments are reported in Table 6. The cosmesis was graded to be “excellent” or “good” for 92.4 %, 90.6 %, and 87.3 % of the treated breasts as reported respectively by the patients at 6, 12, and 24 months, and for 93.6 %, 89.9 %, and 87.6 % of the treated breasts as reported respectively by the surgeons at 6, 12, and 24 months. On a rating scale of 1 (not beneficial) to 4 (very beneficial), the perceived beneficial impact of the BZ on breast cosmesis was rated by the surgeons as a “3” or “4” (moderate to very beneficial impact on cosmesis) for 78.8 %, 77.0 %, and 67.6 % of the treated breasts at 6, 12, and 24 months, respectively.

**Table 6:** Cosmesis evaluation of BioZorb^®^ (BZ). Surgeon and patient survey responses at time of surgery and follow-up assessment^a^

	3-Month FU (0–135 days) (*n* = 425) *n* (%)	6-Month FU (136–270 days) (*n* = 395) *n* (%)	12-Month FU (271–450 days) (*n* = 422) *n* (%)	18-Month FU (451–630 days) (*n* = 259) *n* (%)	24-Month FU (631–900 days) (*n* = 274) *n* (%)
Cosmesis evaluation–patient	(*n* = 385)	(*n* = 380)	(*n* = 403)	(*n* = 252)	(*n* = 268)
Excellent	166 (43.1)	156 (41.1)	186 (46.2)	95 (37.7)	99 (36.9)
Good	198 (51.4)	195 (51.3)	179 (44.4)	120 (47.6)	135 (50.4)
Fair	20 (5.2)	29 (7.6)	35 (8.7)	32 (12.7)	28 (10.4)
Poor	1 (0.3)	0 (0.0)	3 (0.7)	5 (2.0)	6 (2.2)
Cosmesis evaluation–surgeon	(*n* = 419)	(*n* = 391)	(*n* = 417)	(*n* = 258)	(*n* = 274)
Excellent	191 (45.6)	182 (46.5)	192 (46.0)	99 (38.4)	102 (37.2)
Good	203 (48.4)	184 (47.1)	183 (43.9)	125 (48.4)	138 (50.4)
Fair	23 (5.5)	25 (6.4)	39 (9.4)	30 (11.6)	28 (10.2)
Poor	2 (0.5)	0 (0.0)	3 (0.7)	4 (1.6)	6 (2.2)
BZ impact on cosmesis–surgeon	(*n* = 404)	(*n* = 383)	(*n* = 417)	(*n* = 255)	(*n* = 275)
1 (no effect)	36 (8.9)	28 (7.3)	34 (8.2)	21 (8.2)	31 (11.3)
2	52 (12.9)	53 (13.8)	62 (14.9)	50 (19.6)	58 (21.1)
3	203 (50.2)	202 (52.7)	226 (54.2)	123 (48.2)	143 (52.0)
4 (very beneficial)	113 (28.0)	100 (26.1)	95 (22.8)	61 (23.9)	43 (15.6)

*FU* follow-up

^a^Categorical data are reported as number (%). If more than one visit occurred within an FU interval, the more conservative assessment was chosen for cosmesis

## Discussion

In this first large-scale, multicenter study of patients implanted with the BZ during BCS, we report good oncologic outcome through the midterm follow-up visit and a low rate of reoperations or AEs after BCS. The patient-reported outcomes through 24 months support a significant cosmetic benefit, with a majority reporting good-to-excellent breast cosmesis at all time points. The surgeons’ desire to coordinate surgical findings with ROs was the primary motive behind use of the BZ, with surgeons reporting its use as a communication tool for radiation oncology in 91.4 % of cases.

Classically, ROs define the tumor bed by evidence of surgery, seroma formation, incision position, and surgical clips (if placed). Currently, many breast surgeons are using oncoplastic surgical techniques, which may place incisions in remote locations, create dissecting planes, and alter tissue orientation in the service of enhancing the final cosmetic results.^44^ These newer procedures rearrange neighboring tissue that move into the cancer excision site, often causing seroma formation in a wide area unrelated to the extent of cancer. The typical landmarks used by ROs to define and target the tumor bed are therefore largely obliterated.

Accurate identification of the tumor bed is difficult even when traditional techniques such as placement of surgical clips are used due to the inevitability of some clip migration. From the viewpoint of the RO, with the conformational changes of the breast from a lying-down position to a rolled position, surgical clips may be in very different positions, suggesting that they have migrated. The relative position of the clips to each other is not maintained, raising the risk of inadequate coverage. To compensate for this, ROs tend to increase the PTV with larger margins to account for the uncertainties of patient movement and physiologic variations.^45^ Even if surgeons do not believe clips actually migrate, a target identified by multiple clips moving independently of each other creates uncertainty in targeting.

In an effort to allow ROs to focus better on treatments such as boost and APBI, surgeons may enhance their communication with ROs regarding the accurate location of the original tumor site. Modern radiation oncology uses CT-based treatment planning, and the titanium clips in each BZ are readily apparent on CT.

With the BZ, the cavity of the excised tumor bed is sutured to the device itself. In that way, tissue of the tumor bed remains in contact with the BZ regardless of patient position. With this method, the relative orientation of the lumpectomy margins is maintained regardless of breast position or degree of surrounding tissue rearrangement. The RO then has 3D clarity of the tumor bed from any direction, in contrast to non-cancer-related surgical alterations, thus facilitating exclusion of oncoplastic or rearrangement seromas not related to cancer. Ultimately, this enhanced degree of communication should result in decreased radiation volumes, avoiding increased radiation volumes which have been negatively associated with breast cosmesis.^22^,^24^,^26^

This study demonstrated the usefulness of BZ for focusing radiation treatment planning. The ROs considered the BZ useful for reducing PTV volumes in 46.2 % of the cases for which this was an applicable question. For the ROs who said BZ was useful, the most commonly approximated volume reduction was a 30 % PTV reduction. This corresponds with previously reported study findings from a retrospective cohort study and a case-control study, both of which compared patients with and without BZ placement, and both reporting slightly more than a 30 % reduction in PTV or tumor bed volume for patients implanted with the BZ.^37^,^38^

The ROs assessed the BZ as “very useful” for 86.6 % of the patients receiving APBI, with 85 % of the ROs reporting a 20 % to 30 % decrease in PTV for these patients. This may be expected given the necessity of precise targeting with APBI.

Regarding boost treatment specifically, when asked “Did BioZorb^®^ improve your accuracy in boost targeting?” the ROs responded “yes” in 87.4 % of the cases. This underscores the value of BZ for the patients receiving APBI, for whom precise targeting of the tumor bed is important. Of the 59 patients receiving APBI with available surgical follow-up (FU) evaluation (median FU, 12.7 months), no local or distant recurrence was observed, and good-to-excellent cosmesis was reported for 96.6 % of the breasts by the surgeons and patients. Further study and long-term oncologic outcome would be useful in assessing the particular utility of the BZ for patients receiving APBI.

Cosmetic outcome immediately after BCS often is superior to that observed at subsequent follow-up visits several months after surgery, partly because of progressive RT changes and eventual seroma absorption exposing the loss of natural breast contour. Our study demonstrated consistently good-to-excellent cosmesis per patient-reported outcomes, with only a minimal decrease seen from the 6-month (92.4 %) to the 24-month (87.3 %) patient assessment. Surgeons agreed, rating the BZ as having a moderate to strong effect on cosmesis in a majority of cases (67.6 % to 78.9 %, dependent on time point).

A minimal number of patients considered their cosmesis to be poor, with only 14 patients (1.7 %) reporting poor cosmesis at any time point, and 5 of the 14 patients marking their cosmesis as improved at later follow-up assessments. Informally, the participating surgeons stated that using the BZ as a scaffold gave them the ability to resect wider surgical margins and still be able to restore preoperative symmetry. This is consistent with results reported by Kaufman et al.,^40^ who described long-term (2 to 3 years post-surgery) maintenance of breast contour after BCS and RT using the BZ in a comparison of the treated breast quadrant with the untreated breast quadrant.

The reoperation rates observed in our study (8.1 % re-excision rate and 2.6 % mastectomy rate) compare favorably with those reported by several recent population-based studies in the United States, which found reoperation rates ranging from 21.6 to 30.9 %.^46^^–^48 No post hoc analysis was performed to ascertain why the reoperation rates were lower in our study, which may or may not have been attributable to the BZ. One factor may have been the broad use of oncoplastic techniques, which have lower reported re-excision rates.^49^,^50^ Moderate-to-extensive oncoplastic tissue arrangement was performed for 55.8 % of the breasts, with minor mobilization in 40.7 % of the cases. We theorize that surgeons excised greater volumes due to foreknowledge of volume replacement with the BZ.

Findings have shown reoperation rates to be lower with oncoplastic surgery (OPS) in recent years. A 2017 review of 13 studies comparing OPS and conventional BCS found re-excision rates ranging from 0 % to 18 % (median, 2.7 %) for OPS compared with rates ranging from 0 % to 32 % (median, 13 %) for conventional BCS.^51^ Rates for conversion to mastectomy have ranged from 0 % to 24 % (median, 11.9 %) for OPS versus 1.5 % to 34 % (median, 14 %) for conventional BCS. The re-excision rates across our 14 registry centers ranged from 0 % to 19.8 % (median, 4.9 %), and the rates for conversion to mastectomy rates have ranged from 0 to 6.3 % (median, 1.5 %). Re-excision surgery also may have been affected by the consensus guidelines on margins released in 2014.^52^

We observed a 30-day breast infection rate of 0.5 % and a 90-day rate of 1.3 %. The 30-day value is representative of surgical BZ placement, whereas the later rates include effects of radiation and use of chemotherapy, both of which had an impact on infection development. During the course of the study, only 5 (29 %) of the 17 patients with breast infection required BZ removal, suggesting that either some infections did not involve the device or the device may tolerate infection without requiring removal. The overall breast infection rate during the period of the study was 2.1 %. The reported rates are in line with those commonly observed in BCS, suggesting no elevation of infection risk with BZ implantation.

In a National Surgical Quality Improvement Program (NSQIP) analysis of 30-day complications observed across 226,899 patients undergoing surgery for breast cancer from 2005 to 2017, Jonczyk et al.^53^ reported a 30-day infectious complication rate of 0.5 % to 1.2 % for BCS, dependent on treatment subgroup. An analysis of 23,001 women undergoing BCS between 2004 and 2011 found a 90-day surgical-site infection rate of 1.8 %.^54^ We have found that as long as the device is handled according to guidelines with scrupulous sterile technique and not implanted in patients who are poor candidates for any kind of implant, BZ implantation at the time of BCS should not increase the risk of infection.

The BZ is designed to maintain an intact 3D structure for 1 year or longer so that radiation given after chemotherapy still will have a structural target. The exact time of bioabsorption is difficult to predict, but all devices progressively dissolve (except for titanium clips). Device palpability on follow-up breast examinations was not a collected data point in the registry, without which we cannot contribute meaningfully to the literature on the range of time it may take for the device to resorb. This is a particular item of interest among surgeons because time to resorption can vary considerably across individuals. It is important that patients be made aware of this variation in bioabsorption so expectations can be managed.

A recent single-center study of 89 patients implanted with the BZ found that the BZ still was palpable at the time of the last clinical breast exam (performed at a median 1.1 years) for 63.6 % of the patients, with palpability observed to persist as late as 2.8 years after implantation.^36^ Follow-up datapoints collected in the registry with relevance to device palpability are limited to the event-reporting of pain, which occurred for 1.1 % of the patients and was concentrated in the first 3 months after BCS, with reported reasons for device removal. Only two patients (0.2 %) requested device removal due to pain or anxiety.

For the majority of the investigators involved in the registry, this study constituted their initial experience with the BZ. In our experience, the learning curve for device-sizing was quite steep for the first few cases, particularly if the device-sizer set was not used. An observed early tendency was to select excessively large devices with an aim to fill the lumpectomy cavity. However, this can lead to increased palpability and breast tenderness. The optimal device size should mimic the excised tumor. Appropriate patient selection, device-sizing, adequate device tissue coverage, and tissue closure should minimize any problematic long-term palpability. Chiefly, it is always necessary to mobilize breast tissue to cover the device and not place it directly under the skin. The minimum amount of tissue required for coverage varies, depending on the location of the cavity, the relative size of the cavity compared with the size of the breast, and the availability of surrounding breast parenchyma, which in turn relates to the appropriate-size device chosen, whether it be a conventional style or a low-profile device.

Careful consideration should be given for patients with a smaller breast volume because device palpability is naturally more likely in these patients, particularly if the tumor is large, located in an inner quadrant, or especially superficial or peripheral. However, the more recently available low-profile design of the device should be suitable for many of these superficial lesions. The low-profile device became available later in the study, so study data on its usage were not formally collected.

As a registry study, our investigation was limited by an observational study methodology. Because all clinical decisions were entirely per the discretion of the operating surgeon or RO, significant variance was potentially introduced, but we found that our findings were largely homogeneous. For some RT data points, RT characteristics were available for less than half of the enrolled patients. Because the study primarily enlisted surgeons, associated ROs may or may not have been able to contribute study data. Follow-up data were not collected on device palpability, a datapoint of particular interest to some practitioners. However, very few devices were removed over time for palpability, suggesting that bioabsorption of the device and/or incorporation into tissue was acceptable to the vast majority of patients.

We were further limited by the lack of a comparator arm, which would have been particularly beneficial for comparative assessment of breast cosmesis, although the practicality of accumulating a comparator arm makes this opportunity unlikely. Furthermore, the surgeons, ROs, and patients were assessed after surgical breast cosmesis. All three groups were less objective in assessing cosmesis than a third party would have been, although some studies have identified patient-reported cosmetic outcome as the most valuable measurement of post-BCS breast cosmesis.^55^,^56^ Finally, we were limited by a relatively short median follow-up period of 18 months for this report. Therefore, we could not address whether oncologic outcome and recurrence rates are influenced by BZ usage.

## Conclusion

This is the first large multicenter study assessing outcomes for 818 patients implanted with a 3D bioabsorbable tissue marker during BCS with median follow-up period of 18.2 months. Radiation oncologists found that the device aids in targeting for tumor bed boost and APBI and often is associated with reduced PTVs. Both the patients and surgeons reported good to excellent cosmetic outcomes after BCS with BZ implantation through 24 months. Adverse effects, including 30-day breast infections (0.5 %) and reoperations after BCS with BZ, occurred at acceptably low rates. This multicenter study suggests that the BioZorb^®^ 3D tissue marker is a safe adjunct for BCS and may add welcome benefits with use by both surgeons and ROs.

## Electronic Supplementary Material

Below is the link to the electronic supplementary material.

Supplementary material 1 (DOCX 13 kb)
