# Supplementary material for: A Three-Dimensional Bioabsorbable Tissue Marker for Volume Replacement and Radiation Planning: A Multicenter Study of Surgical and Patient-Reported Outcomes for 818 Patients with Breast Cancer
Source: Ann Surg Oncol. 2020 Nov 21;28(5):2529–42. doi: 10.1245/s10434-020-09271-2 (PMC8043870; doi:10.1245/s10434-020-09271-2)
Supplement: Supplementary file 1 — Supplementary material 1 (DOCX 13 kb) [file 10434_2020_9271_MOESM1_ESM.docx]

| **Supplemental Table 1.** Full registry inclusion and exclusion criteria |
| --- |
| **Inclusion criteria:** |
| 1. Patient must be a female at least 18 years of age at the time of surgery and implantation of BioZorb |
| 1. Patient must read and understand fully the informed consent to participate in the registry |
| 1. Patient must be a candidate for breast excision surgery |
| 1. Patient must be deemed by surgeon to be eligible for marking of the surgical excision site |
| **Exclusion criteria:** |
| 1. Patients who are non-English speaking and therefore cannot give proper informed consent |
| 1. Men with breast cancer |
| 1. Patients presenting with Paget's disease of the nipple |
| 1. Patients with a previous history of breast cancer in the same breast |
| 1. Patients that have already been treated with radiation in the same breast and precludes the use of additional radiation of that breast |
| 1. Patients who are pregnant, lactating, or may become pregnant during treatment |
| 1. Patients with a history of collagen vascular disease such as systemic lupus, scleroderma, etc., that puts them at a high risk for fibrosis and complications |
| 1. Patients with an increased risk for surgical complications such as wound infection (evidence of clinical or subclinical infection at the time of surgery, history of heavy smoking, poorly controlled diabetes, etc.) |
| 1. Patients with two or more breast cancers in different quadrants of the same breast |
| 1. Patients who have sub-glandular breast implants |
| 1. Patients who have electronic implantable devices such as pacemakers or defibrillators |
| 1. Patients who are contraindicated for surgery due to co-existing complex medical factors such as anti-coagulant use which cannot be safely reversed |
| 1. Patients with psychiatric or other conditions that would complicate the informed consent process or delivery of care |
